# Supplementary material for: Management of thyroid eye disease: a Consensus Statement by the American Thyroid Association and the European Thyroid Association
Source: Eur Thyroid J. 2022 Dec 8;11(6):e220189. doi: 10.1530/ETJ-22-0189 (PMC9727317; doi:10.1530/ETJ-22-0189)
Supplement: Supplementary Table 1: Conflict of Interest Disclosures ATA-ETA Thyroid Eye Disease Consensus Statement. [file supplementary_table_1.pdf]

**Supplementary Table 1:** Conflict of Interest Disclosures ATA-ETA Thyroid Eye Disease Consensus Statement

| First Name | Last Name | Financial Disclosure Form? | FDF Date Received? | Disclosures Noted | Is this relationship relevant? | Mitigation Measures |
|---|---|---|---|---|---|---|
| Henry | Burch | Yes | 12/08/2020 | UpToDate | No | |
| Petros | Perros | Yes | 11/03/2020 | IBSA Institut Biochimie | No | |
| Tomasz | Bednarczuk | Yes | 11/04/2020 | Merck<br>Berlin-Chemie<br>Novartis<br>Ipsen<br>Roche diagnostics | No<br>No<br>No<br>No<br>No | |
| David | Cooper | Yes | 3/31/2020 | None | | |
| Peter | Dolman | Yes | 12/16/2020 | Immunovant | No | |
| Angela | Leung | Yes | 05/10/2021 | Vertice<br>Medscape<br>China Merck<br>IBSA Institut Biochimie | No<br>No<br>No<br>No | |
| Ilse | Mombaerts. | Yes | 11/05/2020 | None | | |
| Mario | Salvi | Yes | 11/29/2020 | IBSA Institut Biochimie<br>Valenza-Bio<br>Roche - Institutional Funding | No<br>No<br>No<br>Yes | Recusal from discussion of teprotumumab, institutional support was considered non-exclusionary. |

| | | | | | | |
|---|---|---|---|---|---|---|
| | | | | only<br><br>Horizon-institutional support only | | |
| Marius | Stan | Yes | 10/16/2020 | Horizon-institutional support only<br><br>Immunovant<br><br>Siemens Healthineers<br><br>Tolmar | Yes<br><br>No<br><br>No<br><br>No | Recusal from discussion of teprotumumab; institutional support was considered non-exclusionary. |
