# Supplementary material for: Management of thyroid eye disease: a Consensus Statement by the American Thyroid Association and the European Thyroid Association
Source: Eur Thyroid J. 2022 Dec 8;11(6):e220189. doi: 10.1530/ETJ-22-0189 (PMC9727317; doi:10.1530/ETJ-22-0189)
Supplement: Supplementary Figure 1: Objective eye measurement in TED. [file supplementary_figure_1.pdf]

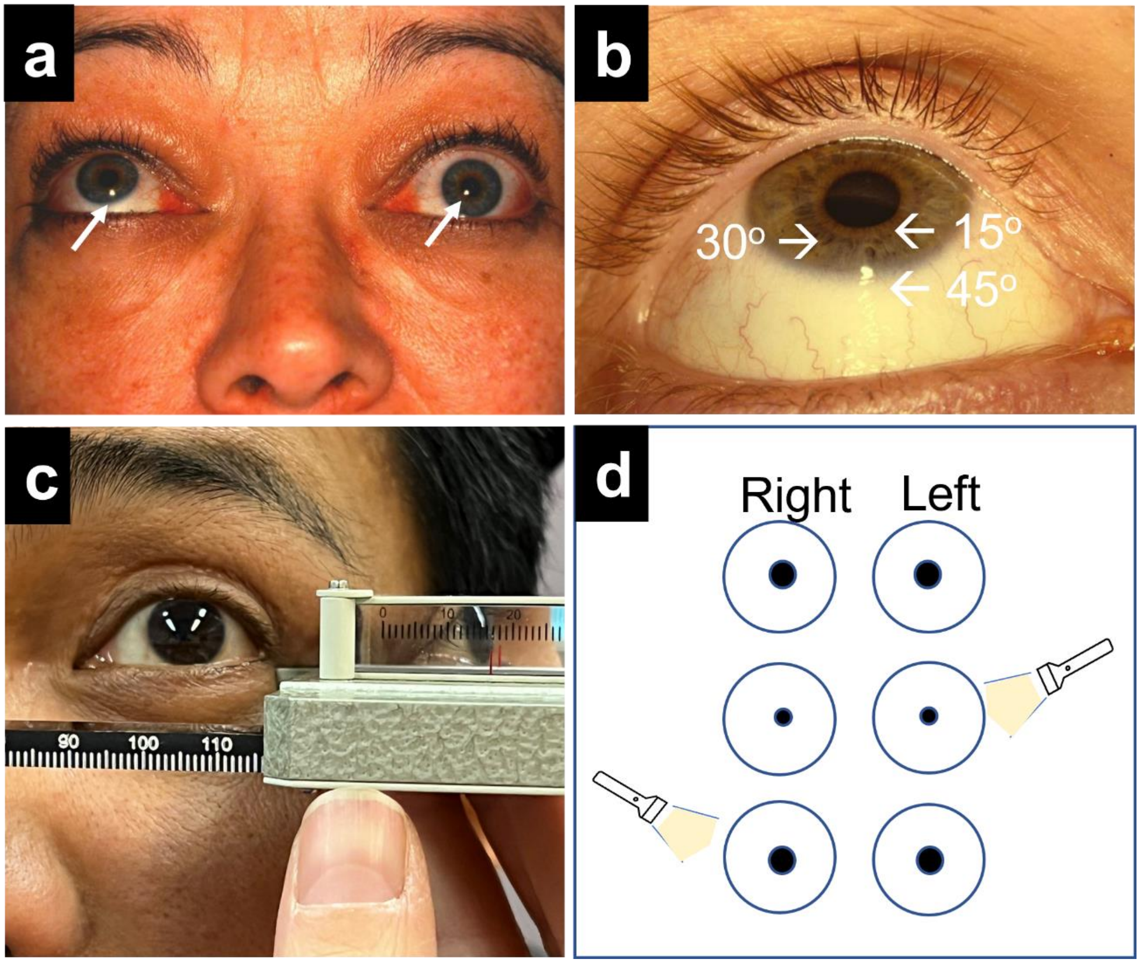

Supplementary Figure 1: Objective eye measurement in TED

a, b: Estimating ocular ductions with corneal light reflex: The examiner shines a penlight on the patient's eyes and observes the light reflex on the cornea as the patient is instructed to look in 4 directions, up, down, right, and left (Courtesy of P Dolman).

a. Light reflexes on cornea (white arrows) as patient attempts upgaze. Right light reflex is midway between limbus and pupil edge, so eye is elevating 30°. Left eye reflex is inside pupil and upgaze limited to 10°

b. Right eye looking up: Light reflects from the limbal edge meaning the patient has full duction of 45°. If upgaze is limited so that the light reflects from the pupil edge, the patient duction is restricted to 15° and if it is seen midway between the pupil edge and limbus, the duction is restricted to 30°.

c. Exophthalmometer allowing protrusion of eye to be measured through the prism (Courtesy P Dolman).

d. Swinging light test: Patient with right afferent pupillary defect. Penlight shone on left normal eye elicits a full bilateral pupillary constriction. Penlight shone on right eye with optic nerve conduction deficit elicits a lesser pupillary constriction, causing apparent relative dilatation of both pupils (courtesy of H Burch).

Abbreviations: TED: thyroid eye disease
