# Supplementary material for: Management of thyroid eye disease: a Consensus Statement by the American Thyroid Association and the European Thyroid Association
Source: Eur Thyroid J. 2022 Dec 8;11(6):e220189. doi: 10.1530/ETJ-22-0189 (PMC9727317; doi:10.1530/ETJ-22-0189)
Supplement: Supplementary Figure 2: Surgical outcomes in TED (Courtesy P Dolman). [file supplementary_figure_2.pdf]

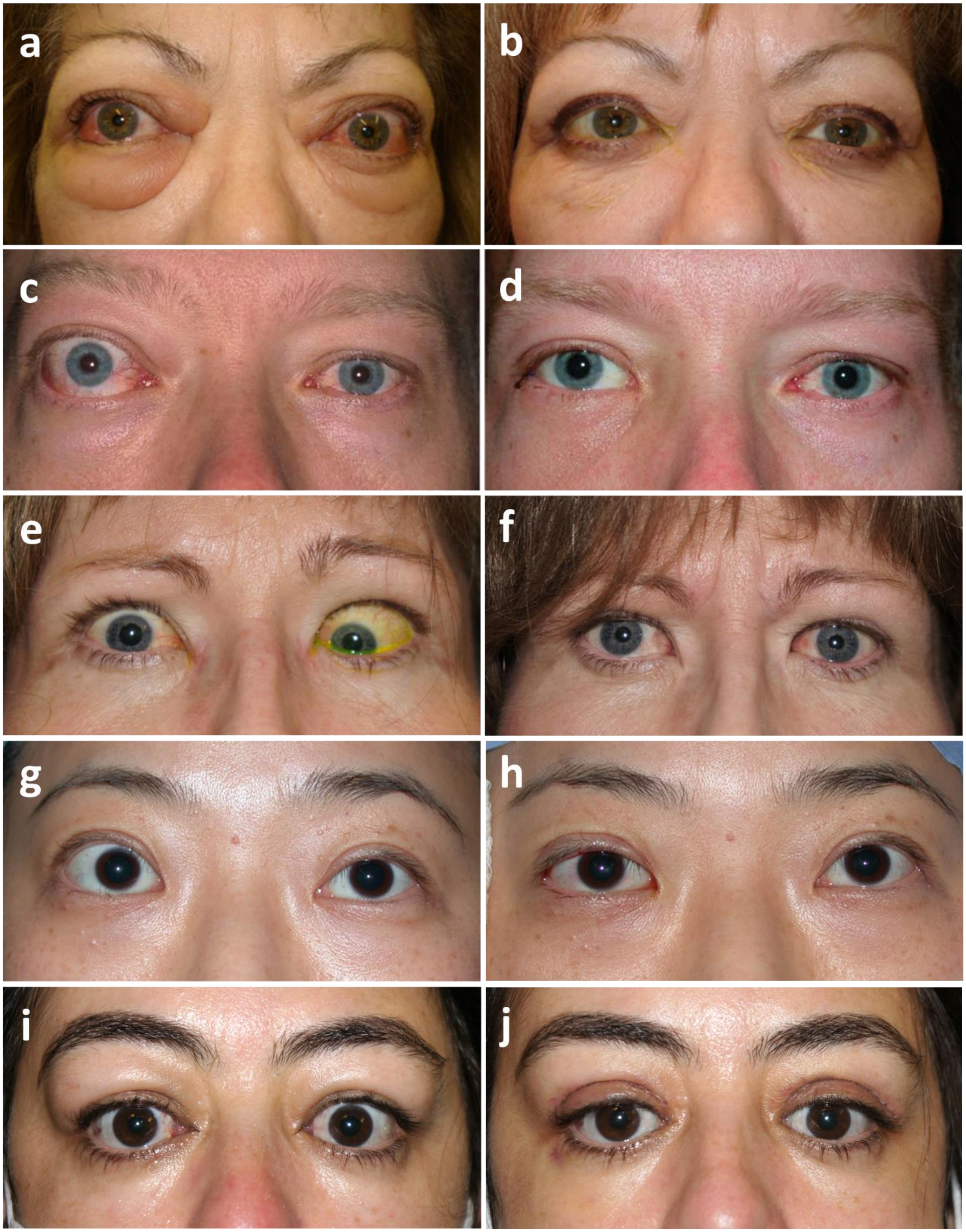

Supplementary Figure 2: Surgical outcomes in TED (Courtesy P Dolman)

a, b. Congestive orbitopathy: a**:** This patient had persistent periocular inflammatory signs with congestion and edema that was non-responsive to IVGC. She had no progression in any of her severity parameters for over 6 months. Although her CAS scores were very high, she had inactive disease (false positive CAS). b**.** 2 Weeks

following orbital decompression, her soft tissue inflammatory signs had markedly improved (CAS reduced to zero) from reduction in venous congestion rather than disease inactivation.

c: Right asymmetric proptosis from fat expansion with lid retraction.

d. Improved symmetry following right orbital decompression for disfiguring proptosis along with upper eyelid lowering and lower eyelid elevation.

e, f: Quiescent orbitopathy with misaligned eyes, strabismus, and retracted eyelids, before and after ocular alignment and upper eyelid lowering surgery.

g, h: Isolated right upper eyelid retraction before and after levator recession surgery from a posterior approach

i, j: Bilateral upper lid retraction and upper lid and sub-brow fat expansion before and after upper lid lowering from an anterior approach along with fat sculpting blepharoplasty.

Abbreviations: CAS: clinical activity score; IVGC: intravenous glucocorticoids; TED: thyroid eye disease
