# Supplementary material for: Management of thyroid eye disease: a Consensus Statement by the American Thyroid Association and the European Thyroid Association
Source: Eur Thyroid J. 2022 Dec 8;11(6):e220189. doi: 10.1530/ETJ-22-0189 (PMC9727317; doi:10.1530/ETJ-22-0189)
Supplement: Supplementary Figure 3: Diagnostic criteria and suggested office-based examination by endocrinologists for assessment of TED. [file supplementary_figure_3.pdf]

Diagnostic criteria for TED[a]

Eyelid retraction present + any of the following:

- -thyroid dysfunction
- -proptosis
- -dysthyroid optic neuropathy
- -EOM (double vision or restricted eye movements)

Eyelid retraction absent + thyroid dysfunction + any of the following:

- -proptosis
- -optic nerve dysfunction
- -extraocular muscle involvement

All other presentations

- -consider alternative diagnoses

Examination

Patient is seated relaxed with head in the primary gaze position and focusing in the distance

1. Lids
   a. Look for swelling and redness of upper and lower lids (A)
   b. Look for upper and lower lid retraction[b] (B)
   c. Ask patient to shut their eyes gently and look for lagophthalmos (C)

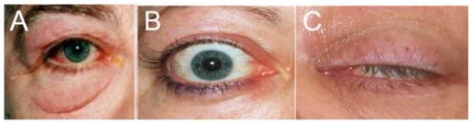

2. Conjunctivae
   a. Look for redness (D) and chemosis (E)

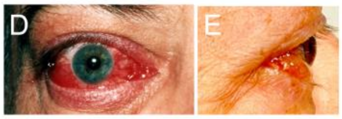

3. Proptosis
   a. Look from either side or from above and note position of cornea in relation to an imaginary line that joins the brow and cheekbone; if the anterior part of the cornea crosses the imaginary line, the patient is likely to have proptosis (F)
   b. Use exophthalmometer (if available and if examiner is trained in its use)
   c. Note position of lower lid[c] (B)

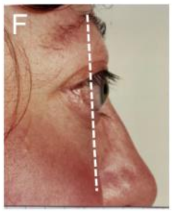

4. Eye movements
   a. Look for abnormal head posture (head tilt backwards or sideways suggests significant EOM restriction) (G)
   b. Look for obvious squint (H)
   c. Ask the patient to move their eyes in the six directions of gaze and observe for restriction, or squint.
   d. Ask the patient if they experience double vision or retro-orbital pain induced by eye movements

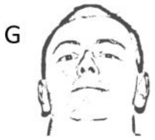

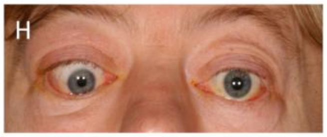

5. Cornea
   a. Look for obvious corneal opacification[d] (I)

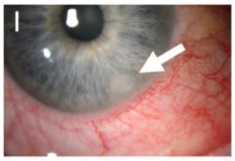

6. Vision[e] (assessment is only required if the history suggests visual loss)
   a. Check corrected visual acuity (small print or Snellen chart if available)
   b. Check for colour desaturation (ask the patient to look at a red dot against a white background in good ambient illumination conditions that apply to the field of vision of both eyes, and ask to view the target with one, then the other eye and ask if there is a difference in brightness of quality of the color)
   c. Check for visual fields defects using the confrontation technique
   d. Check for RAPD (J)
   e. Check for papilledema / optic atrophy

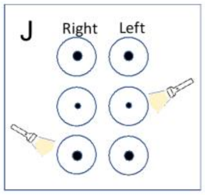

Supplementary Figure S3: Diagnostic criteria and suggested office-based examination by endocrinologists for assessment of TED

Footnotes Figure S3:

[a]From Bartley GB, Gorman CA. Diagnostic criteria for Graves' ophthalmopathy. Am J Ophthalmol. 1995 Jun;119(6):792-5.

[b]Upper lid retraction may be a manifestation of thyrotoxicosis

[c]There is a correlation between lower lid retraction and proptosis and if present is collateral evidence for proptosis

[d]Very rarely seen in an endocrine clinic

[e]Dysthyroid optic neuropathy is rare and an unlikely cause for visual problems in the absence of significant EOM restriction or double vision

Images A, and D with permission from publisher taken from Dickinson AJ, Perros P. Controversies in the clinical evaluation of active thyroid-associated orbitopathy: use of a detailed protocol with comparative photographs for objective assessment. Clin Endocrinol (Oxf). 2001 Sep;55(3):283-303. Images E, F, G and I with patients consent (courtesy of P Perros), B, C and H with patient consent (courtesy of P Dolman)

Abbreviations: DON: dysthyroid optic neuropathy; EOM: extraocular muscle; RAPD: relative afferent pupillary defect
